# Effects of zoledronic acid and the association between its efficacy and γδT cells in postmenopausal women with breast cancer treated with preoperative hormonal therapy: a study protocol

**DOI:** 10.1186/s12967-014-0310-2

**Published:** 2014-11-25

**Authors:** Eriko Sumi, Tomoharu Sugie, Kenichi Yoshimura, Harue Tada, Takafumi Ikeda, Eiji Suzuki, Yoshimasa Tanaka, Satoshi Teramukai, Akira Shimizu, Masakazu Toi, Nagahiro Minato

**Affiliations:** Department of Clinical Innovative Medicine, Institute for Advancement of Clinical and Translational Science, Kyoto University Hospital, 54 Shogoin Kawahara-cho, Sakyo-ku, Kyoto 606-8507 Japan; Breast Surgery, Kansai Medical University Hirakata Hospital, Osaka, Japan; Center for Clinical Research, Kobe University Hospital, Hyogo, Japan; Department of Data Science, Institute for Advancement of Clinical and Translational Science, Kyoto University Hospital, Kyoto, Japan; Department of Experimental Therapeutics, Institute for Advancement of Clinical and Translational Science, Kyoto University Hospital, Kyoto, Japan; Department of Breast Surgery, Kyoto University Hospital, Kyoto, Japan; Center for Therapeutic Innovation, Graduate School of Biomedical Sciences, Nagasaki University, Nagasaki, Japan; Department of Biostatistics, Graduate School of Medical Science, Kyoto Prefectural University of Medicine, Kyoto, Japan; Department of Immunology and Cell Biology, Graduate School of Medicine, Kyoto University, Kyoto, Japan

**Keywords:** Zoledronic acid, Postmenopausal women, Breast cancer, γδ T cells, Letrozole

## Abstract

**Background:**

Although the efficacy of zoledronic acid in postmenopausal women with breast cancer has been suggested, the underlying mechanism has not been fully clarified. Therefore, which patients may benefit from zoledronic acid and the optimal frequency of zoledronic acid administration are unclear. This study evaluates the effects of zoledronic acid on the tumor response in postmenopausal women with breast cancer and explores the relationship between its efficacy and γδ T cells.

**Methods/design:**

This study is an open-label, multi-institutional, single-arm, phase II clinical trial. Zoledronic acid will be administered once during preoperative hormonal therapy with letrozole for 24 weeks in postmenopausal women with Estrogen Receptor (ER)-positive , Human Epidermal Growth Factor Receptor 2 (HER2)-negative, clinical T1 or T2 N0M0 breast cancer. The primary endpoint is the objective response rate measured by MRI at 12 and 24 weeks. The secondary endpoints are the associations between the frequency of Vγ2Vδ2 T cells before the administration of zoledronic acid and the objective response, the association between the frequency of Vγ2Vδ2 T cells and the Preoperative Endocrine Prognostic Index score, and the association between the frequency of Vγ2Vδ2 T cells and Ki67 (MIB-1 index).

**Discussion:**

This study is designed to determine the add-on effect of zoledronic acid during preoperative hormonal therapy and to investigate the changes of the frequency of Vγ2Vδ2 T cells after the administration of zoledronic acid to explore the potential mechanism of zoledronic acid in breast cancer patients.

**Trial registration:**

This trial was registered at the UMIN Clinical Trials Registry as UMIN000008701.

## Background

According to the 2008 National Breast Cancer Registry Report [1], more than half of breast cancers are Estrogen Receptor (ER)-positive, Human Epidermal Growth Factor Receptor 2 (HER2)-negative, less than 5 cm in diameter and negative for lymph node metastasis. Nevertheless, treatment with paclitaxel and anthracycline-based combinations as an adjuvant therapy in ER-positive, HER2-negative patients is less effective [2].

Hormonal therapy for early breast cancer has been shown to be effective [3]. Anastrozole is as effective as tamoxifen as a neoadjuvant treatment in postmenopausal women [4,5]. Letrozole is also more effective than tamoxifen as a preoperative treatment in postmenopausal patients with ER- and/or Progesterone Receptor (PgR)-positive primary breast cancer, although the response rate of 55% [6] is suboptimal.

Recently, the addition of zoledronic acid to adjuvant endocrine therapy in premenopausal women with breast cancer receiving goserelin improved disease-free survival and reduced the risk of disease-free survival events overall [7]. Moreover, the rates of both disease-free survival and overall survival were improved by the addition of zoledronic acid in postmenopausal patients [8,9].

Zoledronic acid appears to be efficacious in patients with early breast cancer [7-9] and in those with low levels of estrogen or a deficiency of estrogen [7-9] after menopause or oophorectomy and who were receiving hormonal therapy [7,8].

The mechanism of the efficacy of zoledronic acid seems to be through direct anti-tumor effects [10]. Many theories and hypotheses have been presented on the mechanisms of zoledronic acid on tumor response [11-13]. The involvement of immune cells, such as Vγ2Vδ2 T cell (also called γδ T cells), has also been suggested [14]. Zoledronic acid is one of the nitrogen-containing bisphosphonates (N-BPs). N-BPs, such as zoledronic acid and pamidronate, inhibit farnesyl diphosphate synthase, and its upstream metabolite diphosphomonoesters (e.g., isopentenyl diphosphate) accumulate in tumor cells. Then, Vγ2Vδ2 T cells specifically recognize and lyse the sensitized target tumor cells that bear or express antigenic substances [15,16]. The activated Vγ2Vδ2 T cells also secrete cytokines, such as interferon-γ (IFN-γ) [17]. Although it was demonstrated that the frequency of γδ T cells in peripheral blood was increased and that an acute-phase reaction occurred after treatment with pamidronate [18], the causal relationship between the function of γδ T cells and the efficacy of pamidronate has not been elucidated.

The hypothesis in this study is that zoledronic acid will elicit an anti-tumor effect in patients with early breast cancer who have a high frequency of γδ T cells in the peripheral blood. In addition, the therapeutic roles of γδ T cells might be determined by measuring body temperature and IFN-γ production because γδ T cells secrete inflammatory cytokines in response to antigenic stimulation through their T cell receptors. The aim of this study is to examine the mechanism underlying the add-on effect of zoledronic acid and to determine whether the initial frequency of γδ T cells is related to the objective response rate of patients with early breast cancer who receive hormone therapy with zoledronic acid.

### Objectives

The main objective of this study is to examine the add-on effect of zoledronic acid in the treatment of postmenopausal women with ER-positive, HER2-negative breast cancer who receive preoperative hormonal therapy by letrozole. Other objectives are as follows: the determination of the frequency of Vγ2Vδ2 T cells in peripheral blood before the administration of zoledronic acid, the analysis of the associations between the frequency of Vγ2Vδ2 T cells before the administration of zoledronic acid, the objective response, the association between the frequency and Preoperative Endocrine Prognostic Index score, and the association between the frequency and Ki67 (MIB-1 index).

## Methods/design

### Study design

This study is an open-label, multi-institutional, single-arm, phase II clinical trial. A total of 75 women will be enrolled after they give written informed consent. The study was approved by the ethical committee at Kyoto University on October 18, 2012 (C-646) and will be approved by the ethical committees at the participating hospitals prior to study initiation at each site. The study protocol complies with the Helsinki declaration [19] and the Ethics Guidelines for Clinical Research of the Ministry of Health, Labor, and Welfare [20]. This study was registered with the UMIN Clinical Trials Registry as UMIN000008701 (http://www.umin.ac.jp/ctr/index.htm). An additional study to investigate tumor infiltration with γδ T cells at baseline, 12 weeks and 24 weeks was also approved by the ethical committee at Kyoto University on June 1, 2013 (E1723).

### Eligibility criteria

Women will be included in the study if they meet the following criteria: have primary cT1-2, N0M0 breast cancer; histologically confirmed invasive ductal cancer; ER-positive and HER2-negative (i.e., as determined by either immunohistochemistry (IHC) or by FISH/DISH (fluorescence *in situ* hybridization/Dual Color *in situ* Hybridization)) breast cancer; low levels or a deficiency of estrogen (i.e., women aged 60 years or older that have been postmenopausal for more than 4 years or have had a post-bilateral oophorectomy); no prior treatment for breast cancer; measurable disease on enhanced MRI within 6 weeks prior to study entry; an Eastern Cooperative Oncology Group (ECOG) performance score of 0 or 1; are within the age range of 20 to 75; have adequate renal function with a Ccr (Cockcroft-Gault) > = 30 mL/min; and have adequate hepatic function with serum bilirubin < = 2.0 and AST/ALT < = 100.

Women will be excluded if they have: hyperparathyroidism that requires management; uncontrolled diabetes mellitus; diseases that require continuous management with systemic corticosteroids; dental and/or periodontal disease that requires invasive treatment after enrollment; currently active or past malignancies within the past five years; prior bisphosphonate therapy, if they have undergone hormone replacement therapy within 7 days prior to enrollment; are hypersensitive to contrast materials for MRI; or used other investigational drugs within 28 days prior to enrollment.

### Intervention

Participants will take 2.5 mg/day of letrozole orally within 7 days of enrollment in the study. Participants will receive a single-drip injection of zoledronic acid on day 28. The dose of zoledronic acid will be reduced when the participants demonstrate impaired renal function (i.e., when the Ccr is equal to or less than 60 mL/min) according to Table 1. A single administration of zoledronic acid is expected to be effective because frequent zoledronic acid treatments lead to a decrease in blood Vγ2Vδ2 T cell levels [14]. Cytokines such as IL-2 are not used in combination with zoledronic acid because patients with early breast cancer without any previous treatment have a potential to restore immune responses against tumor antigens without administration of cytokines such as IL-2. Participants will take letrozole every day for 24 to 26 weeks until they have a mastectomy as a part of routine medical care. The study treatment will be terminated upon disease progression, episodes of serious adverse events, or in patients for whom zoledronic acid cannot be administered within 35 days of starting letrozole treatment.

**Table 1:** **Dose of zoledronic acid**

	**Ccr (mL/min)**			
	**>60**	**50 - 60**	**40 - 49**	**30 - 39**
Dose of zoledronic acid	4 mg	3.5 mg	3.3 mg	3.0 mg

### Measurements

Patients will be evaluated clinically and through laboratory testing, radiological testing and pathological testing, according to Table 2.

**Table 2:** **Trial schedule**

**Protocol treatments and assessments**		**Pre-enrollment**	**Week 1**	**Week 4**	**Week8**	**Week 12**	**Week 16**	**Week 20**	**Week 24**	**Mastectomy**
**Protocol treatments**	**Letrozole**									
	**Zoledronic acid**			↓						
Demographics, current medications, dental examination		○								
Physical examination		○		○^1^						
Complete blood count, blood biochemical test, bone metabolism markers		○		○^2^	○		○		○	
Enhanced MRI, tumor markers		○				○			○	
Palpation, ultrasound examination		○		○	○	○	○	○	○	
Vδ1+ T cells and Vδ2+ T cells in peripheral blood ^3^				○	○				○	
IFN- γ ^4^				○						
Pathological examination^5^		○			○					○
PEPI score										○

^1^Body temperature before and 4–6 hours after the administration of zoledronic acid.

^2^Bone metabolism markers that are expected.

^3^The frequency of Vδ1+ T cells and Vδ2+ T cells among CD3+ T cells in peripheral blood.

^4^IFN-γ at 4–6 hours after the administration of zoledronic acid.

^5^ER, PgR, Ki67.

The primary endpoint is the objective response rate (ORR). The ORR is defined as the proportion of the complete response (CR) and the partial response (PR) within the best overall response based on MRI evaluation. Patients will undergo tumor assessments at baseline, 12 weeks and 24 weeks by investigators using the Response Evaluation Criteria in Solid Tumors (RECIST) version 1.1 [21]. The imaging modality is restricted to MRI because MRI is superior to other modalities for the detection and staging of invasive breast cancer, and MRI measurements after neoadjuvant treatment are more highly correlated with pathological tumor size than mammography or ultrasound [22]. The secondary endpoints are the change in tumor volume, ORRs using ultrasonography or clinical assessment, breast-conserving surgery, the frequency of Vγ2Vδ2 T cells, PEPI score [23], Ki67 (MIB-1 index) and safety.

Tumor volume will be measured using MRI volumetry at baseline, 12 weeks and 24 weeks by an independent committee. The contrast-enhanced MRI will be performed according to the European Society of Breast Imaging (EUSOBI) guidelines [24]. ORRs using ultrasonography or clinical assessment will be evaluated separately by the investigators according to RECIST. A partial resection of the breast will be regarded as breast-conserving surgery after the completion of letrozole treatment.

The frequency of Vδ1^+^ T cells and Vδ2^+^ T cells among CD3^+^ T cells in peripheral blood mononuclear cells will be analyzed by two-color flow cytometry [11] and will be evaluated at baseline, 4 weeks and 20 weeks after the administration of zoledronic acid.

Peripheral mononuclear cells (PBMC) will be purified by density gradient centrifugation. For flow cytometric experiments, 2 × 10^5^ cells of PBMC in each well will be stained with mAbs. The following mAbs will be used: fluorescein isothiocyanate (FITC)-conjugated anti- Vδ1 mAbs (Thermo Scientific, Rockford, IL), Vδ2 mAbs (Beckman Coulter Inc., Fullerton, CA), anti-CD3 mAbs (BioLegend, San Diego, CA), phycoerythrin (PE)-conjugated anti-CD3, CD4, CD8, CD25, NKG2D mAbs (BD Biosciences, San Diego, CA) and FcR blocking reagent (Miltenyi Biotec GmbH, Bergisch Gradbach, Germany) will be used. The stained cells will be subjected to 2-color flow cytometry using a FACSCalibur flow cytometer (Becton–Dickinson, Franklin Lakes, NJ) and the analyses will be performed on a single sample. The serum at 5 hours after administration of zoledronic acid will be collected and subjected to ELISA for IFN-γ level using an ELISA Kit (PEPROTECH, Rocky Hill, NJ) according to the manufacturer’s instructions. The analyses will be conducted on triplicate samples.

Adverse events will be evaluated according to the Common Terminology Criteria for Adverse Events (CTCAE) version 4.0 [25].

### Data analysis

The sample size was determined using the Bayesian method based on predictive distributions [26,27]. The ORR of preoperative hormonal therapy by letrozole is estimated to be 45% [28]. The threshold ORR of 45% was used as the prespecified target value for the primary endpoint. The uniform distribution and the degenerated distribution at an expected ORR of 60% were used as the analysis prior and the design prior, respectively. The sample size of 75 is required to preserve the Bayesian power of 80% with a prespecified probability threshold of 95% to declare the treatment efficacious.

The primary statistical analysis will be performed using the Bayesian approach according to the intention-to-treat principle. The posterior beta distribution of the ORR will be calculated using the prior uniform distribution. If the posterior probability that the ORR is greater than the prespecified target value of 45% exceeds a prespecified probability threshold of 95%, the treatment will be considered efficacious.

A subset analyses for the ORR based on MRI and tumor volume using MRI volumetry according to (a) equal and greater or less than the median of the frequency of Vγ2Vδ2 T cells before the administration of zoledronic acid, (b) equal and greater or less than the median of IFN-γ at 5 hours after the administration of zoledronic acid and (c) presence of fever and/or flu-like symptoms, are planned. Exploratory subgroup analyses for the frequency of Vγ2Vδ2 T cells according to (a) equal and greater or less than a body mass index of 25, (b) 0, 1–3, or over 3 of PEPI score, (c) equal and greater or less than 14 of Ki67, (d) equal and greater or less than the median of IFN-γ at 5 hours after the administration of zoledronic acid and (e) presence of fever and/or flu-like symptoms, are also planned.

## Discussion

We presented the design of a single-arm phase II study to evaluate the add-on effect of zoledronic acid in preoperative hormonal therapy. The single-arm design will not clarify whether the frequency of Vγ2 T cells before administration of zoledronic acid influences the outcome of breast cancer patients regardless of zoledronic acid or if they influence the outcome related to administration of zoledronic acid. Nevertheless, the changes of the frequency of Vγ2Vδ2 T cells after the administration of zoledronic acid suggest the potential immunological mechanism of zoledronic acid in breast cancer patients.

### Trial status

Ongoing.

## Abbreviations

ER: Estrogen receptor
HER2: Human epidermal growth factor receptor 2
N-BPs: Nitrogen-containing bisphosphonates
IFN-γ: Interferon-γ
PEPI: Preoperative endocrine prognostic index
IHC: immunohistochemistry
FISH: Fluorescence *in situ* hybridization
DISH: Dual color *in situ* Hybridization
ECOG: Eastern cooperative oncology group
ORR: Objective response rate
CR: Complete response
PR: Partial response
RECIST: Response evaluation criteria in solid tumors
EUSOBI: European society of breast imaging
CTCAE: Common terminology criteria for adverse events
